# Decoding oral leukoplakia: microbiome dysbiosis and inflammatory dynamics unveiled in a rat model

**DOI:** 10.3389/fmicb.2025.1613165

**Published:** 2025-10-22

**Authors:** Zhiqin Sang, Yufeng Zhang, Enoch Kao, Tingting Zhu, Jiazhen Yang, Zhenjiang Zech Xu, Shi Huang, Fei Teng, Wanchun Wang

**Affiliations:** ^1^Qingdao Stomatological Hospital Affiliated to Qingdao University, Qingdao, China; ^2^Hospital of Ocean University of China, Qingdao, China; ^3^Faculty of Dentistry, The University of Hong Kong, Hong Kong, Hong Kong SAR, China; ^4^State Key Laboratory of Food Science and Technology, Nanchang University, Nanchang, China

**Keywords:** oral leukoplakia (OLK), 2bRAD-M, oral mucosal microbiota, immune factors, animal model

## Abstract

**Introduction:**

Oral leukoplakia (OLK) is an oral precancerous lesion associated with oral microbiome dysbiosis and systemic inflammation. However, the longitudinal changes of the microbiome and its causal relationship with inflammation remain unclear, and traditional sequencing struggles to detect low-biomass samples.

**Methods:**

A 4-nitroquinoline-1-oxide (4-NQO)-induced rat OLK model was used. The oral microbiome was analyzed via 2bRAD-M sequencing; serum levels of tumor necrosis factor-α (TNF-α) and interleukin-6 (IL-6) were measured. Additionally, functional pathway analysis of the microbiome and its correlation with inflammation were conducted.

**Results:**

In OLK, we observed significant shifts in the oral microbial diversity, marked by elevated abundances of *Streptococcus*, *Glaesserella*, and *Pseudomonas aeruginosa*. Moreover, shifts in the microbiota precede the manifestation of clinical symptoms of OLK. Functional pathway analysis highlighted enrichment in metabolism, quorum sensing, and cancer-associated microRNA pathways. Serum levels of inflammatory markers (TNF-α and IL-6) were significantly elevated in OLK and significantly correlated with specific bacterial taxa.

**Discussion:**

This study demonstrates the utility of 2bRAD-M sequencing in overcoming traditional metagenomic limitations, offering a high-resolution view of microbiome dynamics in low-biomass environments such as the oral mucosa. These findings establish the oral microbiota as candidate early biomarkers for OLK screening and prevention, opening avenues for precision diagnostics and targeted therapies to mitigate cancer risk associated with OLK.

## 1 Introduction

Oral leukoplakia (OLK) is primarily characterized by the formation of whitish or grayish patches occurring on the oral mucosa that cannot be scraped off and lack histopathological features of other diagnosable conditions. OLK has been widely recognized in the medical field as an oral potentially malignant disorder (OPMD) with a risk of malignant transformation, and its incidence is higher as age increases (Chen et al., 2021). OLK is classified into homogeneous and non-homogeneous types, with the latter having a higher malignant transformation rate (Villa and Sonis, 2018). A seven-year follow-up study revealed that the malignant transformation rate of non-homogeneous leukoplakia exceeded 60% (Abadie et al., 2015). Once OLK undergoes malignant transformation, it significantly reduces patients’ quality of life. Furthermore, OLK often presents with subtle symptoms that are easily overlooked by patients. Conducting in-depth research on OLK can help patients and clinicians identify effective screening and diagnostic methods, improve early detection rates, and create opportunities for timely treatment. The etiology of OLK remains unclear, although it may be associated with local irritants such as smoking, alcohol consumption, and betel quid chewing (Zhu et al., 2021; Randall et al., 2022; Warnakulasuriya and Chen, 2022). In addition to these factors, advancements in sequencing technologies have allowed researchers to explore OLK from a microscopic perspective, revealing associations between OLK and gene mutations (Wils et al., 2023), DNA damage (Mello et al., 2020) and imbalances in the oral mucosal microbiome (Pietrobon et al., 2021).

The oral cavity hosts a complex and diverse microbial community that interacts with the oral mucosal epithelium and immune molecules to maintain oral microecological stability (Lin et al., 2021; Baker et al., 2024). Under normal circumstances, the oral microbiota and the host coexist in a mutually beneficial symbiotic relationship. These microorganisms aid the host in food digestion, nutrient absorption, and maintaining the balance of the oral environment (Sedghi et al., 2021). However, when this homeostasis is disrupted, opportunistic pathogens may have greater chance of proliferation and production of harmful metabolites or virulence factors, thereby causing oral diseases such as dental caries, gingivitis, and periodontitis (Peng et al., 2022; Hajishengallis et al., 2023). More seriously, this dysbiosis can also affect systemic health (Siqueira and Rôças, 2017). With expanding research, it has been suggested that an imbalance in the host’s oral local environment may be closely related to the development of OLK. It is widely accepted that bacteria play a critical role in host mucosal immune responses, and microbial dysbiosis can lead to immune dysfunction in the oral mucosa (Lin et al., 2021; Zheng et al., 2020; Rojas et al., 2022). As OLK is a lesion of the mucosal epithelium, changes in microbial composition and the resulting dysbiosis on the epithelial surface may contribute to epithelial barrier damage and inflammatory responses, hence OLK progression (Saikia et al., 2023).

Based on the aforementioned research premise, in addition to addressing conventional risk factors, monitoring and studying the role of the microbiome in the occurrence and development of OLK have become an important direction for early screening and diagnosis of the condition. Researchers have applied 16S rRNA sequencing to compare the microbiota at OLK lesion sites with corresponding healthy sites in the same individuals and healthy individuals (Amer et al., 2017), revealing significant microbial differences between lesion and healthy sites. Compared to controls, species enriched in OLK lesions include *Rothia mucilaginosa*, *Alloprevotella* sp., *Neisseria meningitides*, *Leptotrichia* sp., *Campylobacter*, and *Rothia* sp. A study by Herreros-Pomares et al. (2021) using 16S rRNA sequencing to compare pathological tissues of proliferative verrucous leukoplakia patients with normal mucosal tissues in healthy individuals found that the microbiota diversity and abundance were higher in healthy individuals. They hypothesized that *Campylobacter jejuni* and *Tannerella* might be significant risk factors for malignant transformation in OLK. Similarly, Decsi et al. (2019) analyzed OLK lesion and healthy tissues using metagenomic sequencing, discovering increased levels of *Fusobacterium nucleatum* and decreased levels of *Streptococcus mitis* in OLK lesions.

However, current studies using metagenomic technologies mostly focus on the association between oral microbiota and OLK, and the results are inconsistent. This inconsistency may be due to the following two main reasons (Pignatelli et al., 2024; Abdul et al., 2024):

1) Cross-sectional experimental design: Clinically, it is relatively easy to obtain samples from healthy controls and OLK patients for comparative analysis of microbial differences. However, such a design not only ignores individual variability but also fails to capture the dynamic changes in the microbiota during the transition from health to disease, making it difficult to uncover the causal relationships and pathogenic mechanisms between oral microbiota and OLK (Hajishengallis et al., 2023). The oral microbiota is a dynamic system (Sedghi et al., 2021; Lamont et al., 2018), and even under the same research conditions, samples collected at different time points may exhibit different characteristics (Bowen et al., 2018).
2) While 16S rRNA amplicon sequencing offers a cost-effective way to obtain a quick overview of microbial communities, it cannot provide the landscape-like profile of oral microbiota associated with OLK (Leggieri et al., 2021). It has several limitations:

- Amplicon sequencing depth typically reaches only the genus level (Bharti and Grimm, 2021), making it challenging to distinguish differences at the species and strain levels. Yet, such high-resolution differences are often critical for disease research (VanEvery et al., 2023).
- It does not provide functional information on the species or strain. Microbes can exhibit “radically different metabolic functions within the same species,” meaning that different strains within the same species may vary significantly in the functional roles of driving the disease development (VanEvery et al., 2023). For example, even if the abundance of a genus is unrelated to a phenotype, the functional pathways of specific species or strains within the genus might be associated with disease. Since oral microbiota can influence diseases through gene regulation changes without altering community structure (e.g., no changes in alpha or beta diversity), functional genes are undoubtedly important for understanding disease mechanisms (VanEvery et al., 2023).
- Amplification bias using different primers. The choice of the 16S rRNA amplified region can significantly affect the conclusions of a study. For example, sequencing results from the V1–V3 regions of a sample differ significantly from those of the V4–V5 regions, reducing comparability across studies (Regueira-Iglesias et al., 2023; Abellan-Schneyder et al., 2021). Additionally, full-length PCR amplification for tissue or low-biomass samples often introduces biases (Knight et al., 2018).
- Bacteria-specific sequencing. Depending on the study target (e.g., bacteria or fungi), different methods such as 16S, 18S, or ITS sequencing are required, preventing comprehensive identification of all targets in a single sequencing run, and suffering from lower sample utilization, increased contamination risk, and raised costs due to multiple sequencing runs. As OLK is a lesion of the mucosal epithelium, changes in the microbial structure and even microbial dysbiosis on the host epithelial surface often occur as a result of epithelial barrier damage and inflammatory responses, thereby influencing OLK progression (Saikia et al., 2023). Recognizing the key microbial pathogens is crucial for activating and precisely regulating the host immune system in disease treatment (Knight et al., 2018). However, existing etiological studies on OLK predominantly focus on either microbes or host factors in isolation, failing to jointly examine the components of microbiome and host immunity and their synergetic interactions that may modulate OLK pathogenesis.

The emerging metagenomics methods provide unique opportunities for studying the microbiological etiology of OLK. Studies on intestinal diseases often utilize rat or mouse models to simulate disease onset and progression, offering a reference for microbial etiology research in oral diseases. 2bRAD-M (He et al., 2023; Sun et al., 2022), a reduced metagenome sequencing technology, enables high-resolution microbiome analysis in low-microbial-biomass and/or host-rich clinical samples, and provides gene function information similar to whole genome sequencing method. It has demonstrated the following specific advantages: (a) simultaneous detection of bacteria, fungi, and archaea; (b) high-resolution microbial identification which can reach the species and strain levels; (c) compatibility with challenging samples (e.g., low-biomass, DNA- degraded, or highly host-contaminated samples) that conventional methods are not capable of analyzing.

In this study, we established a rat model of OLK lesions induced by 4-nitroquinoline-1-oxide (4-NQO) and employed 2bRAD-M to longitudinally track the progression of OLK. By analyzing the association between oral microbiota and OLK progression and measuring the expression of inflammatory factors in tail vein serum using ELISA, this study aims to investigate the etiology and molecular mechanisms of OLK, providing new insights for the early diagnosis and warning of OLK.

## 2 Materials and methods

### 2.1 Study subjects

Adult male Sprague–Dawley (SD) rats (~150 g) were purchased from the Jinan Pengyue Laboratory Animal Breeding Center (Shandong, China). The rats were housed under specific pathogen-free (SPF) conditions at the Laboratory Animal Center of Qingdao University Affiliated Qingdao Stomatological Hospital. They were housed in separate cages and provided standard feed and sterilized drinking water. All drinking water, feed, and bedding materials were sterilized at high temperatures. The animal room was maintained at a constant temperature and humidity, with a relative humidity of 40–70% and a stable temperature range of 18–25 °C. Proper air filtration and ventilation were ensured, along with a simulated natural light–dark cycle of 12 h per day. The animal protocol was reviewed and approved by the Ethics Committee of Qingdao University Affiliated Qingdao Stomatological Hospital (Approval No. 2022KQYX025). All experiments were conducted in accordance with the Guide for the Care and Use of Laboratory Animals.

An oral leukoplakia (OLK) rat model was induced by providing drinking water containing 4-nitroquinoline-1-oxide (4-NQO). Clinical observations and recordings of the dorsal tongue mucosa were conducted biweekly. According to previous experiments by our group, a stable OLK model is established after 8 weeks of feeding. In this study, observation groups were set at different time points: the healthy control group (T0 group, *n* = 8), the 4th-week induction group (T1 group, *n* = 8), and the OLK model group (T2 group, *n* = 8).

### 2.2 Sample collection of oral swabs, serum, and tongue tissues collection of oral swabs

To collect oral swabs, the rats were immobilized in a supine position with their mouths held open passively. Sterile flocked swabs were used to swab the soft and hard tissues of the oral cavity in a specific sequence to ensure comprehensive sampling of all areas. Care was taken to avoid contamination during the process. After collection, the swabs were immediately placed in sterile centrifuge tubes and rapidly frozen in liquid nitrogen. Dynamic environmental sampling was also conducted in the feeding area. All samples were stored at −80 °C with appropriate labeling for subsequent experimental use. Oral swab samples were later analyzed using 2b-RAD sequencing technology.

### 2.3 Collection of tail vein serum

At weeks 0 and 8 of the feeding cycle, three rats were randomly selected for serum collection. The rats were immobilized, and their tails were wiped with alcohol swabs to ensure proper vasodilation. Using sterile scissors, the tail tip (3–5 mm) was quickly trimmed, and the tail was suspended above a blood collection tube containing a coagulation activator. Approximately 0.5 mL of blood was allowed to flow into the tube by gravity. After blood collection, the wound was disinfected, and hemostasis was achieved using a cotton ball. Blood samples were centrifuged at 3,000 rpm for 3 min to obtain serum, which was stored at −20 °C for subsequent enzyme-linked immunosorbent assay (ELISA) analysis.

### 2.4 Collection of tongue tissue

At weeks 0 and 8 of the feeding cycle, three rats were randomly selected and euthanized by an overdose of sodium pentobarbital. The mucosal state of the dorsal tongue was recorded, and tongue tissue samples were collected and immediately immersed in 4% paraformaldehyde for tissue fixation. These samples were later used for hematoxylin and eosin (HE) staining.

### 2.5 2bRAD-M sequencing

Genomic DNA was extracted from the oral swabs of SD rats using a DNA kit according to the manufacturer’s instructions. The integrity of the extracted DNA was verified using 1% agarose gel electrophoresis, and the DNA concentration was measured with the Qubit PicoGreen fluorescence quantification system. To monitor potential background contamination, two blank swabs were processed alongside the samples as negative controls.

Genomic DNA was used to construct sequencing libraries and subjected to 2bRAD-M sequencing. Synthetic primers and 5-NNN-3 adapters were prepared as 100 μM stock solutions and stored at −20 °C. DNA digestion was performed using BcgI enzyme (200 ng DNA, 14 μL enzyme mix) and incubated at 37 °C for 3 h. Digestion efficiency was verified via agarose gel electrophoresis. Digested DNA was ligated with T4 DNA ligation buffer and adaptors at 4 °C for 12 h.

Ligation products were enriched using PCR (20 μL system with primers and 7 μL ligation product), followed by gel electrophoresis and purification of the 100-bp target band. Barcode sequences were introduced via PCR, and products were purified with the QIAquick kit and quantified using Qubit.

Bioinformatic processing of raw reads included quality filtering to remove sequences with a Phred quality score (*Q*-score) <20 or containing >8% ambiguous bases (N-bases). Contaminant sequences derived from environmental or reagent sources were identified and removed using the decontam R package (v1.12.0) with a prevalence-based threshold of *p* < 0.05. The negative control samples showed minimal contamination (≤0.07% of total reads), confirming the reliability of the microbial profiles obtained. Finally, the libraries were sequenced on the NovaSeq 6000 PE150 platform. All raw sequences were deposited in the Sequence Read Archive (Accession ID: SRP437677).

### 2.6 Hematoxylin-eosin staining

Tissue sections were deparaffinized in xylene, hydrated through graded ethanol, and rinsed in water. Hematoxylin staining was performed for 5–15 min, followed by differentiation in 1% hydrochloric acid ethanol for 30 s or until the tissue turned red. Sections were rinsed, blued in a bluing solution, and stained with eosin for 1–3 min. After rinsing, the sections were dehydrated through graded ethanol, cleared in xylene, and mounted with neutral resin. The prepared slides were observed under a light microscope to assess tongue tissue lesions.

### 2.7 Enzyme-linked immunosorbent assay

ELISA plates were equilibrated at room temperature, and wells were set for standards, samples, and blanks. Standards and test samples were added to designated wells, followed by horseradish peroxidase-labeled detection antibodies, except in blank wells. After a 1-h incubation at 37 °C, plates were washed thoroughly to remove unbound materials. Substrates A and B were added to develop color, incubated at 37 °C in the dark, and the reaction was stopped with a stop solution. Optical density (OD) values were measured at 450 nm using a microplate reader, and sample concentrations were determined from a standard curve. Each sample was tested in triplicate to ensure accuracy and reliability.

### 2.8 Bioinformatics and statistical analysis

With 2bRAD sequencing data, we employed the 2bRAD-M computational pipeline^1^ to identify the species-level microbial composition and estimate their abundances by searching against the prebuilt 2b-Tag-DB, a reduced version of GTDB database (v202) and FungiDB database (r48) including the species-unique markers for 45,555 microbial species, 2,339 archaea species and 581 fungal species. Then, we produced the species-level abundance profile for all tissue samples for downstream statistical analysis.

In this study, QIIME2^2^ was used to perform the microbial ecology analysis for oral microbiota, including diversity, community structure, species composition, and functional profiles. R (v4.2.3) and Origin 2021 also utilized for supplementary data analysis and visualization. Alpha diversity metrics, including Chao1, Shannon, and Simpson indices, species richness were computed within samples and compared across samples. The between-sample difference (beta diversity) was computed using unweighted UniFrac distance, with PerMANOVA applied to quantitatively measure the inter-group differences (*p* < 0.05 indicating significance), and *R*^2^ values used to measure the explanatory power of group differences. Functional predictions were made using PICRUSt2 in QIIME2, and results were mapped to the Kyoto Encyclopedia of Genes and Genomes (KEGG) database to identify functional differences at three levels.

TNF-α and IL-6 levels were statistically analyzed using GraphPad Prism 8, with triplicate measurements for each experiment. Spearman correlation analysis evaluated associations between microbiota and inflammatory markers (Benjamini–Hochberg adjusted *p* < 0.2 indicating significance), with positive correlations (*r* > 0) and negative correlations (*r* < 0). Group comparisons employed the Wilcoxon rank-sum test (pairwise) and Kruskal–Wallis test (three groups), with Benjamini–Hochberg FDR correction for multiple comparisons (*p* < 0.05 considered significant).

## 3 Results

### 3.1 Experimental modeling oral leukoplakia in the SD rats

**Figure 1:**
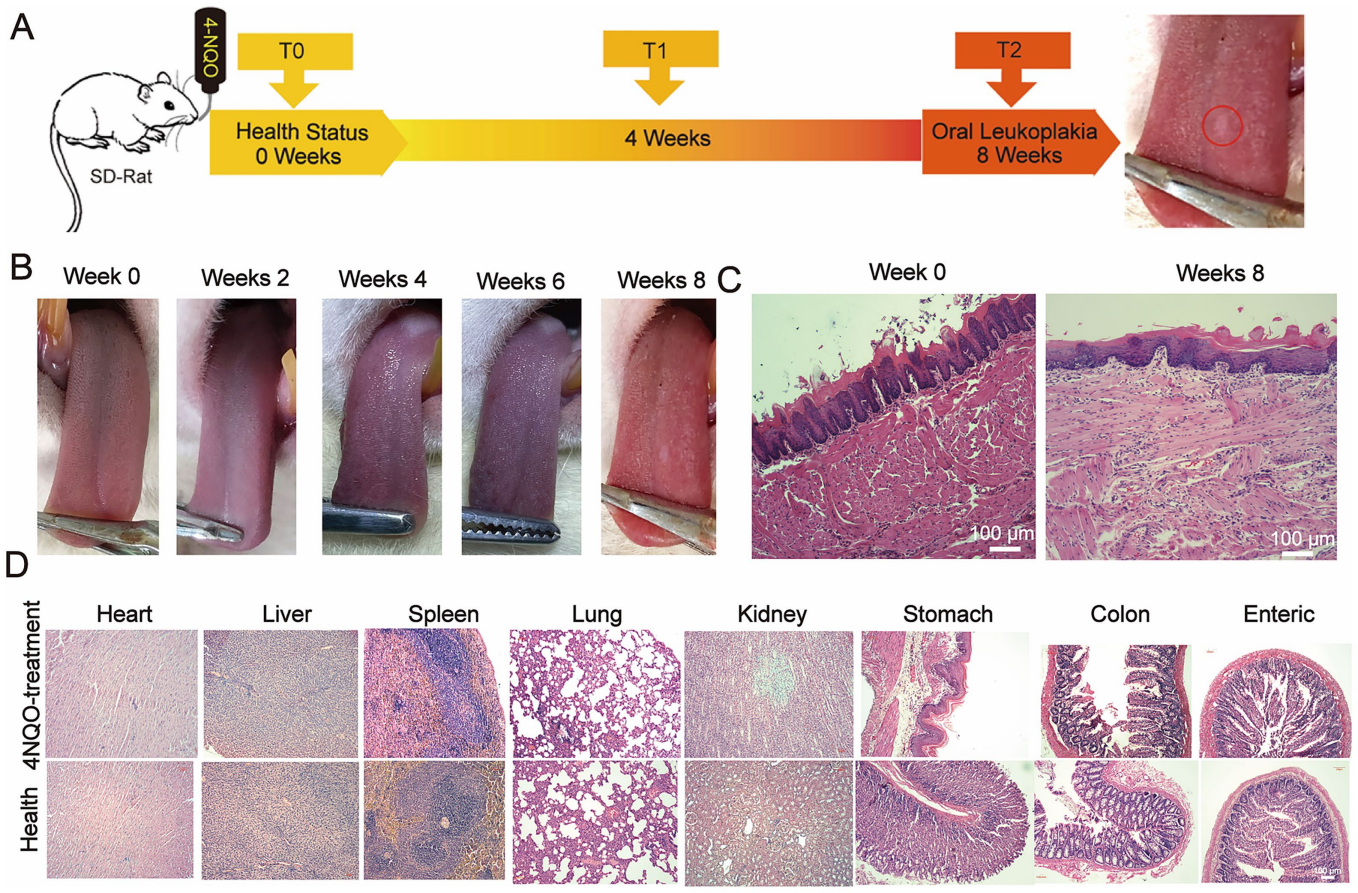
Modeling the OLK on the tongue mucosa in SD rats. **(A)** Schematic of the 4-NQO-induced leukoplakia model in SD rats, showing the administration of 4-NQO-containing drinking water over 8 weeks to induce oral lesions. **(B)** Macroscopic changes in the tongue mucosa of SD rats over 0 to 8 weeks of 4-NQO treatment, illustrating the progression of leukoplakia. **(C)** H&E-stained histological images of the tongue mucosa at baseline (0 weeks) and at 8 weeks post-treatment, highlighting the development of pathological features (scale bar: 100 μm). **(D)** H&E-stained histological images of major organs (e.g., liver, kidneys, lungs) at the end of the 8-week treatment, confirming the absence of significant damage to systemic tissues following model induction (scale bar: 100 μm).

We successfully established a 4-NQO-induced OLK model in SD rats. As shown in Figure 1A, non-wipeable white keratotic plaques were observed on the dorsal tongue mucosa of SD rats exposed to 4-NQO in drinking water by week 8. These white plaques were slightly raised above the mucosal surface (Figure 1B). Hematoxylin-eosin (HE) staining of the tongue lesions at week 8 (Figure 1C) revealed simple hyperplasia of the tongue mucosal epithelium, hyperkeratosis, and parakeratosis with keratin plugs in the keratin layer. Additionally, a small number of neutrophils were observed to aggregate, with a prominent granular layer; hypertrophic spinous cells, and elongated epithelial ridges were consistent with the histopathological features of OLK.

Furthermore, no significant histopathological damage or inflammation was observed in the major organs (heart, liver, spleen, lungs, kidneys, large intestine, small intestine, and stomach) of the modeled SD rats (Figure 1D). These results confirmed the successful establishment of a 4-NQO-induced OLK model in the dorsal tongue mucosa of SD rats, without lesions in other organs.

### 3.2 Oral microbiota alterations during oral leukoplakia development in SD rats

**Figure 2:**
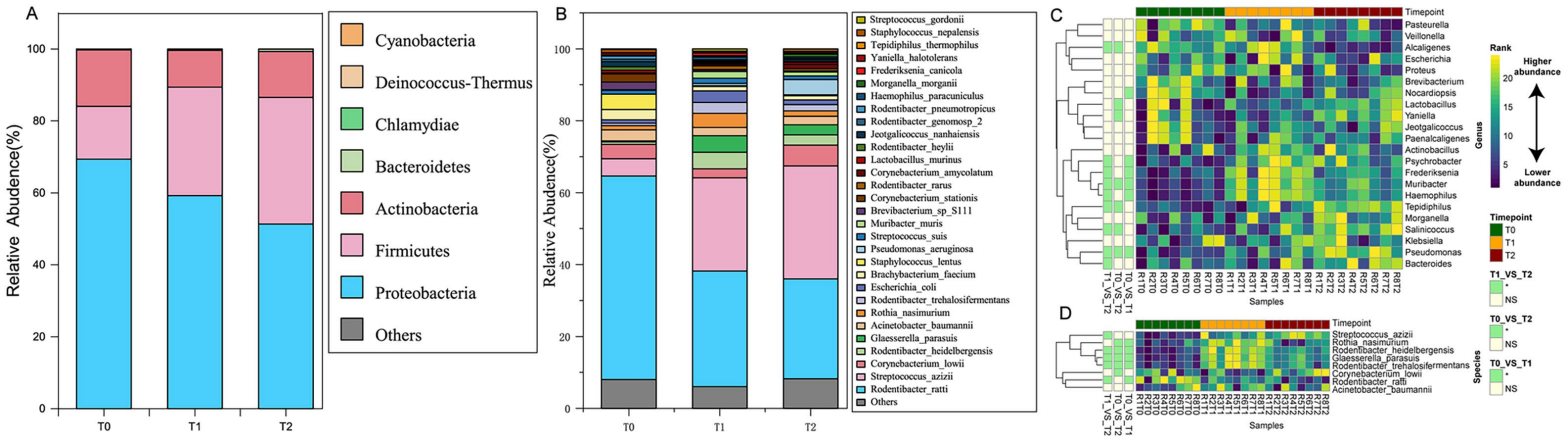
Characteristic species composition in oral microbiota in SD rats at three developmental stages of OLK. **(A)** Cumulative proportion of species composition at the phylum level for the T0, T1, and T2 groups. **(B)** Cumulative proportion of species composition at the genus level for the T0, T1, and T2 groups. **(C)** Heat map showing the microbial taxa at the genus level differentially abundant between at least two time points. **(D)** Heat map showing the species-level taxa differentially abundant between at least two time points.

2bRAD-M sequencing was performed on 24 oral microbiota samples using the NovaSeq 6000 PE150 platform. The BcgI-enzyme-digested DNA fragments were harvested using our computational pipeline (Enzyme_Reads). Reads with more than 8% N bases or low quality were removed, resulting in 213,369,857 high-quality reads. For each sample, Quality_Reads constituted over 80% of Enzyme_Reads on average. These reads identified 16 known bacterial phyla, including 60 classes, 136 orders, 268 families, 1,020 genera, and 4,546 species. Notably, the pipeline also revealed the presence of one archaeal phylum (comprising 3 classes, 4 orders, 4 families, 4 genera, and 6 species) and two fungal phyla (comprising 14 classes, 28 orders, 38 families, 56 genera, and 86 species). At the phylum level (Figure 2A), the composition of samples across the three groups was relatively similar. Over 91.66% of the total species were accounted for by four phyla with the highest relative abundance: Proteobacteria (60%), Firmicutes (26%), Actinobacteria (13%), and Bacteroidetes (0.3%). At the genus level (Figure 2B), the relative abundance of species is mainly distributed across the following eight bacterial genera: *Rothia* (2.2%), *Haemophilus* (1.4%), *Pseudomonas* (1.7%), *Acinetobacter* (2.6%), *Staphylococcus* (2.3%), *Corynebacterium* (6%), *Streptococcus* (22%), and *Rodentibacter* (46%), with the relative abundance of other genera being less than 1%.

**Figure 3:**
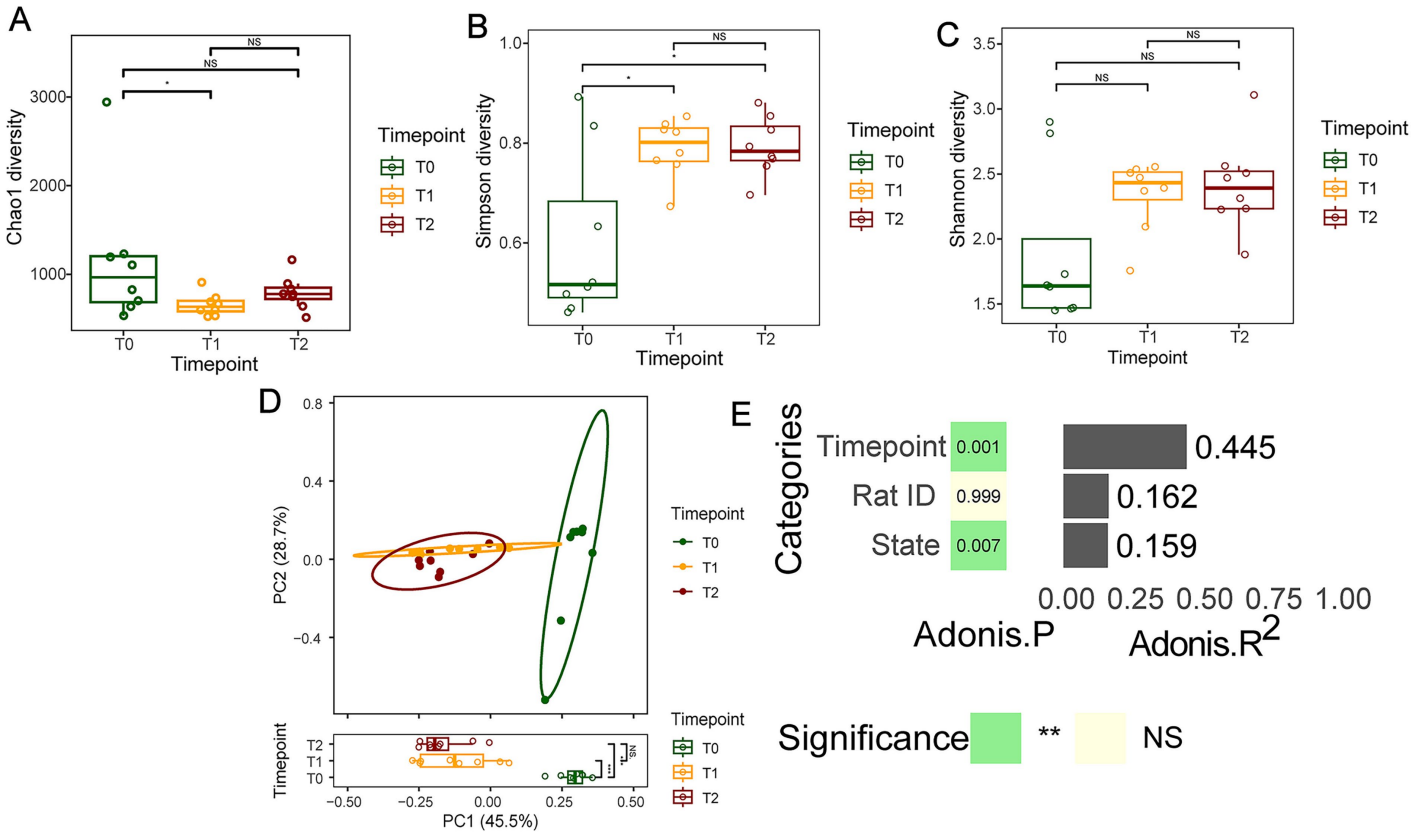
The microbial ecological changes in the oral mucosal microbiota during OLK development of SD rats. **(A)** Boxplots showing the Chao1 index difference among T0, T1, and T2 groups. **(B)** Boxplots showing the difference in Simpson index among T0, T1, and T2 groups. **(C)** The Shannon-diversity distribution in T0, T1, and T2 groups. **(D)** PCoA plot of oral microbiota composition in T0, T1, and T2 groups (*p* = 0.001, *R*^2^ = 0.6691). **(E)** The estimation of effect sizes of co-variates (time point, rat ID, disease state) using Adonis (PerMANOVA).

Additionally, the distance matrix values for T1 and T2 groups were smaller compared to the T0 group (*p* < 0.05, Figure 3), indicating that the oral microbiota structure during OLK development was relatively similar and conserved, with a simpler community structure. In contrast, the community structure was more diverse and complex in healthy oral mucosa.

At the species level, we identified characteristic oral microbial shifts in rats during the OLK progression. By the fourth week (T1 group), compared to healthy rats (T0 group), the relative abundance of six species, including *Streptococcus azizii* and *Glaesserella parasuis*, rose significantly (*p* < 0.05). In contrast, four species, such as *Rodentibacter ratti*, saw a marked decrease in abundance (*p* < 0.05). As OLK fully developed (T2 group), a further transformation occurred in the microbiota. The relative abundance of 10 species, including *Rodentibacter heidelbergensis*, *Streptococcus azizii*, *Glaesserella parasuis*, and *Haemophilus paracuniculus*, increased significantly (*p* < 0.05), while five species, including *Rodentibacter ratti* and *Staphylococcus lentus*, showed a notable decline (*p* < 0.05). Among the species that exhibited an increasing trend, *Streptococcus azizii*, *Glaesserella parasuis*, *Pseudomonas aeruginosa*, and *Corynebacterium amycolatum* stood out (Figures 2C,D).

### 3.3 Alpha diversity alterations during oral leukoplakia development in SD rats

To exploit the impact of OLK development on the within-sample species diversity, a few representative alpha-diversity indices, namely Chao1 richness (total species count), Simpson index (species evenness), and Shannon index (species diversity), were calculated. The Simpson index showed significant differences before and after OLK formation and exhibited an upward trend during OLK progression (*p* < 0.05, Figure 3B). Although there were no significant statistical differences in the Chao1, Shannon and Simpson indices among the three groups, the Chao1 index displayed a significant difference between the T0 and T1 groups (*p* < 0.05, Figure 3A). The Chao index demonstrated a down trend and the others indices demonstrated a gradual upward trend during OLK progression (*p* > 0.05, Figures 3A–C).

To further examine the whole-community-level changes in oral microbiota during OLK development in rats, multivariate statistical analysis was performed using PERMANOVA based on unweighted UniFrac and Euclidean distance matrices. The results revealed that OLK significantly influenced the oral mucosal microbiota community structure (*p* < 0.05, Figure 3). The microbiota of T1 rats showed slight overlap with that of T0 rats (*p* < 0.05), while the microbiota of T2 rats was completely separated from that of T0 rats (*p* < 0.05). However, the oral microbiota of T1 rats was more similar to T2 rats (*p* > 0.05).

### 3.4 Microbial functional changes associated with OLK development

**Figure 4:**
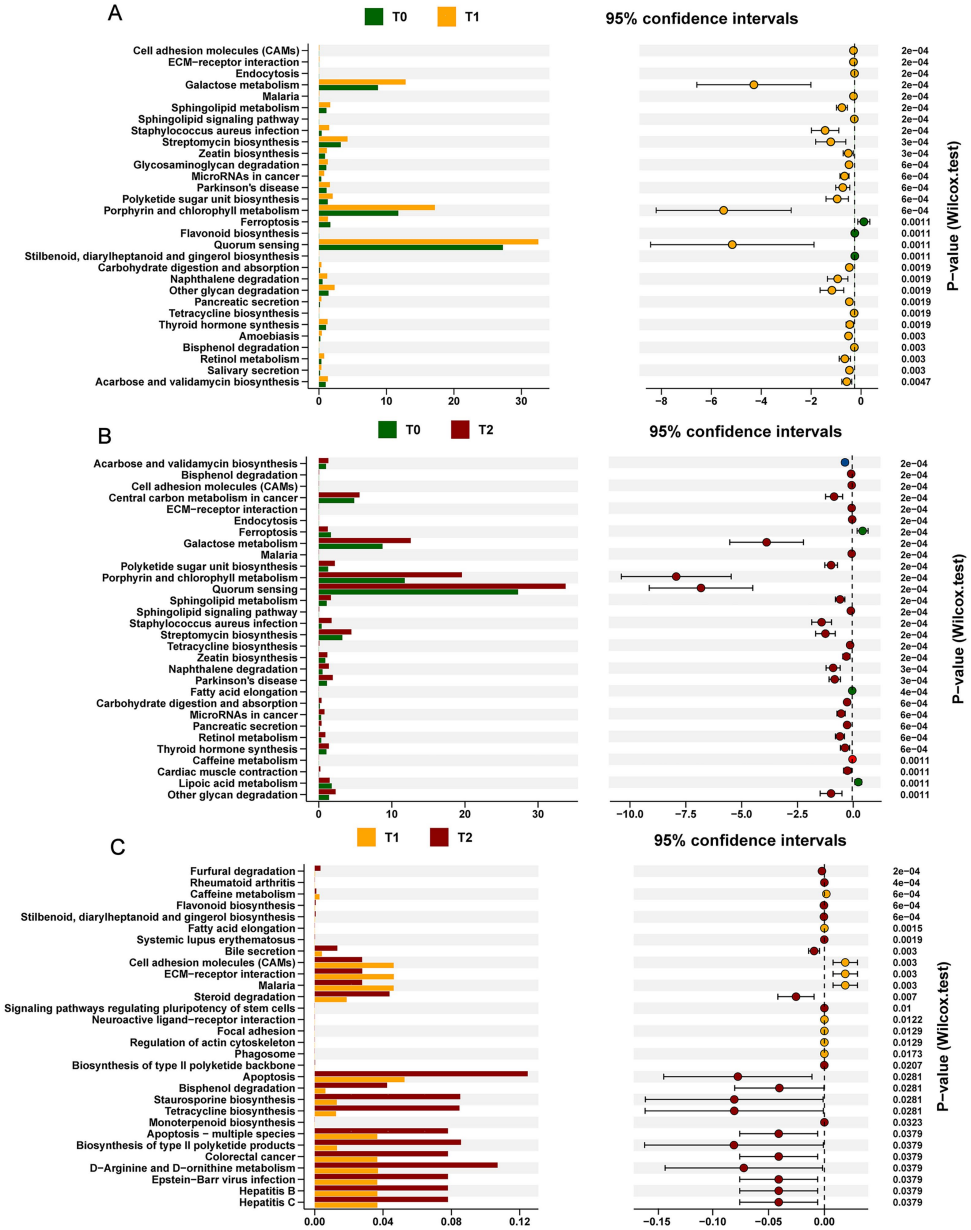
KEGG functional prediction analysis of oral microbiota in SD Rats under OLK development. In the left panel, the bar plots show the mean abundance of functional pathways between two time points. The dot plot with error bars shows the 95% confidence interval of functional difference. **(A)** The KEGG functional pathways differentially abundant at T0 or T1. **(B)** The KEGG functional pathways differentially abundant between T0 and T2 groups. **(C)** The KEGG functional pathways differentially abundant between T1 and T2 groups.

To delve deeper into the functional changes in the oral microbiota during OLK development, we conducted a KEGG analysis of the dynamic shifts in metabolic pathways. Our analysis uncovered 30 metabolic pathways that evolved throughout OLK progression (Figure 4). Notably, compared to the T0 group, we observed significant upregulation of several key metabolism-related pathways during OLK formation (T1 and T2) (*p* < 0.05). These included galactose metabolism within carbohydrate metabolism, ceramide metabolism in lipid pathways, and the corresponding sphingolipid signaling pathways. Perhaps the most intriguingly, the MicroRNA pathways in cancer and bacterial quorum sensing pathways also showed a notable increase (*p* < 0.05), suggesting their potential involvement in OLK progression.

### 3.5 The expression levels of TNF-α and IL-6 before and after OLK in rats

**Figure 5:**
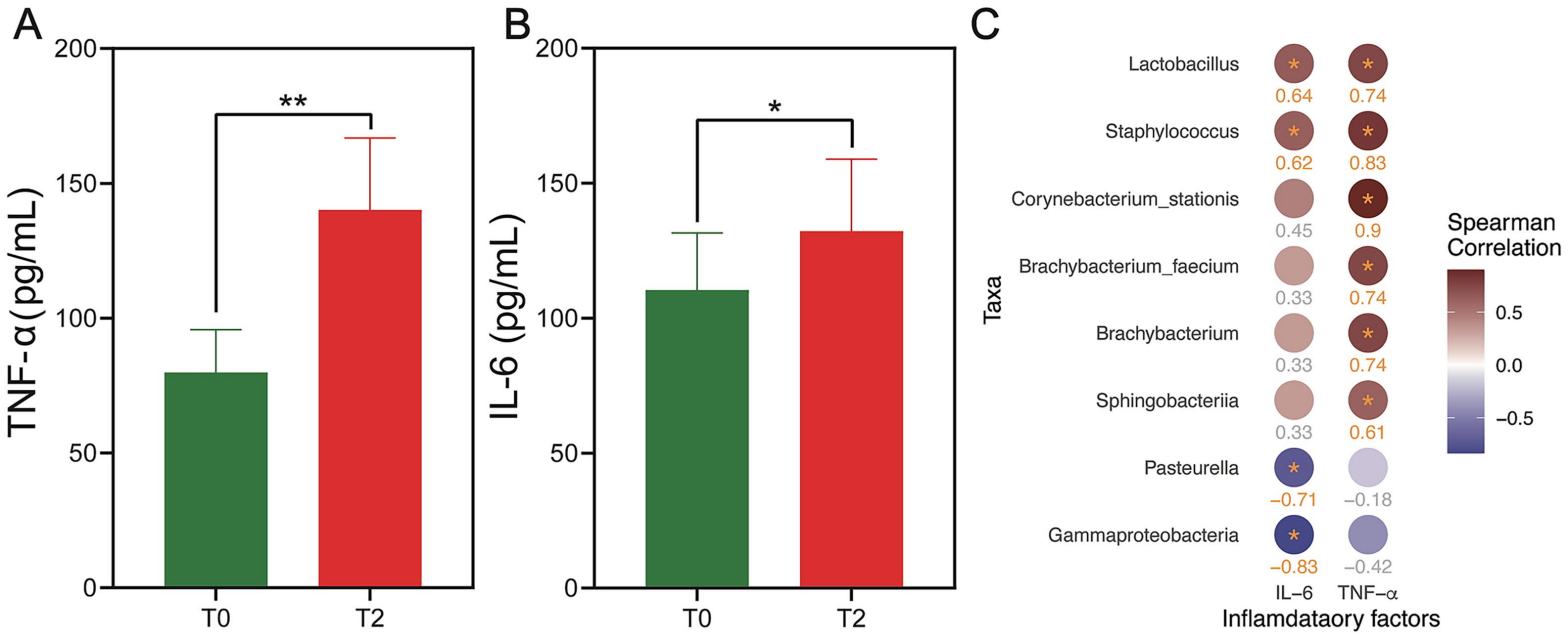
Expression levels of TNF-α and IL-6 in the serum of rats in the T0 and T2 groups and their correlation with key oral microbiota. **(A,B)** The bar plot shows the difference in expression levels of TNF-α **(A)** and IL-6 **(B)** between T0 and T2 groups. The statistical significance is shown as ^**^p < 0.01 and ^*^p < 0.05. **(C)** Heatmap showing the correlation between the microbial taxa (at the phylum, genus, and species levels) and the inflammatory factors (TNF-α and IL-6) at T2. The color of the circles represents the magnitude of the correlation, with red circles indicating positive correlation and blue circles indicating negative correlation. The deeper color, the larger the *r* value. All taxa listed have a statistically significant correlation with *p* < 0.2 (Benjamini–Hochberg adjusted).

To explore the role of inflammation in the progression of OLK, we evaluated serum levels of key inflammatory cytokines, TNF-α and IL-6, in rats at two stages: before (T0) and after (T2) OLK development, using ELISA. As illustrated in Figure 5, rats with OLK (T2) showed a marked elevation in both TNF-α and IL-6 compared to healthy controls (T0), with statistical significance (*p* < 0.05). These findings underscored the pivotal role of inflammation in the pathogenesis of leukoplakia, suggesting that elevated cytokine levels might trigger a cascade of inflammatory responses. This heightened inflammatory milieu could, in turn, interact with microbial shifts, further exacerbating mucosal damage and contributing to the progression of the disease. The intricate interplay between inflammation and microbial dysbiosis warrants deeper investigation to uncover potential therapeutic targets for managing OLK.

### 3.6 Correlation between oral microbiota and inflammation in rats

We conducted the correlation analysis to explore the co-occurrence relationship between species abundance and inflammatory factors (IL-6, TNF-α) in the T0 and T2 groups (Figure 5). The results revealed a significant positive correlation between TNF-α and *Corynebacterium stationis*, *Staphylococcus*, *Lactobacillus*, *Brachybacterium faecium*, *Brachybacterium*, and *Sphingobacteriia* (Benjamini–Hochberg adjusted *p* < 0.2). As for IL-6, we observed a significant positive correlation with *Lactobacillus* and *Staphylococcus* (Benjamini–Hochberg adjusted *p* < 0.2). In contrast, *Pasteurella* and *Gammaproteobacteria* showed a significant negative correlation with IL-6 (Benjamini–Hochberg adjusted *p* < 0.2). No significant correlations were found between species composition and inflammatory factors in the T0 group (Benjamini–Hochberg adjusted *p* > 0.2).

## 4 Discussion

Advances in high-throughput technologies have significantly propelled microbiome research, unveiling the intricate associations between microbial dysbiosis and various diseases (Baker et al., 2017). As the second most abundant microbial habitat in the human body, the oral cavity is home to a complex and diverse microbiota, whose imbalance is closely linked to numerous oral diseases. The microbial shifts within the oral cavity, along with their metabolic by-products, directly influence the host’s immune functions and metabolic responses (Sedghi et al., 2021). Both Gram-positive and Gram-negative bacteria within the oral biofilm possess the ability to alter their phenotypes in response to cell density through a mechanism known as quorum sensing (QS). This cell–cell signaling system enables the coordination of bacterial activities, including biofilm formation and growth, environmental adaptation, competition with potential microbial rivals, and the expression of virulence factors that contribute to pathogenicity (Shao and Demuth, 2010).

Oral leukoplakia (OLK), a common potentially malignant disorder of the oral mucosa, warrants early diagnosis and monitoring, as its timely detection can significantly reduce the incidence of oral cancer (Kabekkodu et al., 2022). Previous studies have demonstrated that the carcinogenic effects induced by alterations in the oral microbiome satisfy many of the hallmarks of cancer, including evasion of growth-suppressive signals and resistance to cell death (Stasiewicz and Karpiński, 2022). Notably, research comparing the oral microbiota of OLK patients with those of oral cancer patients has provided valuable insights into how dysbiosis within the oral ecological niche may heighten the risk of malignant transformation. A 2020 study on periodontitis and oral cancer found that the risk of oral cancer increased in the presence of periodontitis, suggesting a potential link between microbial dysbiosis and cancer progression (Lan et al., 2023). However, the existing studies on microbiome changes in OLK predominantly consist of cross-sectional analyses, which assessed only the disease state at a single time point. These studies are thus limited in their ability to establish a clear connection between temporal microbial shifts and disease progression. Therefore, longitudinal studies are essential for advancing our understanding of the relationship between microbial changes and OLK development.

In this study, we employed 2b-RAD sequencing technology to monitor the microbial dynamics during the progression of OLK in an animal model, providing a comprehensive analysis of microbiome alterations throughout disease development. Animal models serve as an invaluable tool in understanding disease progression and testing therapeutic interventions. Despite significant compositional differences between rodent and human oral microbiomes (Li et al., 2019), rodents remain a widely used and cost-effective model for studying microbial interactions and their impact on health (Hajishengallis, 2023). The compositional differences between rodent and human microbiota do not preclude the rodent model from simulating dysbiotic inflammatory diseases that are also observed in humans. In fact, animal models have long been used to investigate the relationship between oral diseases and microbial changes, such as periodontitis (Ai et al., 2022; Li et al., 2021) and caries (Culp et al., 2021; Huang et al., 2023), thereby enhancing our ability to prevent and treat these conditions. This further supports the notion that although rodents have distinct oral microbiomes, the common microbial features with humans allow them to be confidently employed as disease models.

In line with previous studies, we successfully induced an OLK model in SD rats using 4-NQO and confirmed, through histopathological examination, that the model exhibited pathological changes consistent with those seen in human OLK (Zigmundo et al., 2022). Subsequently, we analyzed the alpha diversity of the oral mucosal microbiota across T0, T1, and T2 groups, revealing a significant reduction in microbial abundance throughout the disease progression. This finding aligns with previous studies where 4-NQO induction in germ-free mice led to a decrease in microbial abundance as oral cancer progressed (Gopinath et al., 2020). In addition, the changes in the Simpson indices means microbial diversity exhibited a general down trend. Importantly, our study established that the microbiota of SD rats with OLK induced by 4-NQO displayed significant structural and diversity differences compared to healthy controls (T0 group).

Beta diversity analysis of the microbial community composition further confirmed that the microbiota of the leukoplakia rats separated distinctly from that of the healthy control group, with the microbial composition of progressing lesions more closely resembling that of the leukoplakia rats. These findings resonate with studies on the salivary microbiome of OLK and oral squamous cell carcinoma (OSCC) patients, which demonstrated substantial overlap between the microbiomes of OLK and OSCC patients, as well as clear distinctions from healthy controls (Gopinath et al., 2020). These results suggest that the oral microbiome of OLK patients may exhibit a shift towards a more pathogenic profile, resembling that seen in oral cancer, further supporting the hypothesis that oral environmental changes, accompanied by microbial dysbiosis, promote disease progression (Sedghi et al., 2021).

While this study successfully documented dynamic changes in the oral microbiome during OLK progression, it is important to acknowledge the differences in microbiome composition between rats and humans, particularly given the presence of gut-derived bacteria in rodent oral microbiomes (Abusleme et al., 2017). Nonetheless, the insights gained from this animal model provide a foundation for future studies that could involve transferring human oral cancer-related microbiota into rodent models, facilitating a deeper understanding of microbial involvement in oral disease progression.

Our findings further revealed that certain bacterial populations, notably *Streptococcus* and *Rothia*, exhibited significantly increased relative abundances in OLK rats compared to healthy controls. This aligns with previous studies linking the overabundance of *Streptococcus* species to the development of various cancers, including oral cancer (Flemer et al., 2018; Zhou et al., 2022). Interestingly, *Rothia*, known for its ability to produce acetaldehyde, was also found to be abundant in the oral cavity of OLK patients (Amer et al., 2020), and acetaldehyde has been implicated as a carcinogenic compound contributing to oral and gastrointestinal cancers (Nieminen and Salaspuro, 2018; Tuominen and Rautava, 2021). Our results suggest that these bacteria, through their metabolic by-products, may play a role in promoting malignant transformation within the oral mucosa.

Moreover, analysis of inflammatory cytokines in the serum of leukoplakia rats revealed significantly elevated levels of TNF-α and IL-6, further supporting the hypothesis that microbial dysbiosis triggers inflammatory pathways that contribute to tissue damage and disease progression. KEGG functional predictions indicated upregulation of sphingolipid metabolism and associated signaling pathways, both of which have been implicated in inflammatory diseases like periodontitis (Lu et al., 2023). Sphingolipids, including ceramide and sphingosine-1-phosphate (S1P), are known mediators of cellular responses, including inflammation and cell death, suggesting that these pathways may play a pivotal role in the pathogenesis of OLK.

In conclusion, our study highlights the dynamic shifts in the oral microbiome during OLK progression, providing new insights into the microbial underpinnings of this potentially malignant disorder. By identifying key bacterial taxa and inflammatory pathways associated with disease progression, this research contributes to the growing body of evidence supporting the role of microbial dysbiosis in oral pathologies and carcinogenesis. Further studies, particularly those involving human microbiota transplantation into rodent models, will be crucial in elucidating the exact mechanisms through which microbial shifts contribute to disease progression and malignancy in the oral cavity.

## 5 Limitations and future directions

While this study provides valuable insights into the dynamic changes of the oral microbiome during OLK progression, several limitations should be acknowledged. First, the sample size (*n* = 8 per group), though consistent with many preclinical studies, may limit the generalizability of the findings. Future studies with larger cohorts are warranted to enhance statistical robustness and validate the observed trends. Second, although the 4-NQO-induced rat model effectively recapitulates key pathological features of human OLK, inherent differences between rodent and human oral microbiomes—including the presence of gut-associated bacteria in rodents—may affect the translational relevance of the results. Nevertheless, the model remains a valuable tool for probing microbial dynamics in a controlled setting.

To build upon this work, we propose several future research priorities: (Chen et al., 2021) validation of these microbial signatures in longitudinal human OLK cohorts to confirm their clinical relevance; (Villa and Sonis, 2018) utilization of gnotobiotic animal models colonized with human-derived oral bacteria to establish causal relationships between specific microbes and malignant progression; and (Abadie et al., 2015) integration of multi-omics approaches (metagenomic, metatranscriptomic, and metabolomic analyses) to elucidate functional mechanisms underlying microbial-driven pathogenesis. These efforts will help bridge the gap between observational associations and mechanistic understanding, ultimately contributing to improved early detection and intervention strategies for OLK.

## Data Availability

The raw sequences presented in this study are publicly available. This data can be found here: https://www.ncbi.nlm.nih.gov/sra, accession number SRP437677.

## Footnotes

^1^ https://github.com/shihuang047/2bRAD-M

^2^ https://qiime2.org/
